# A GDSL-motif Esterase/Lipase Affects Wax and Cutin Deposition and Controls Hull-Caryopsis Attachment in Barley

**DOI:** 10.1093/pcp/pcae041

**Published:** 2024-04-26

**Authors:** Chiara Campoli, Mhmoud Eskan, Trisha McAllister, Linsan Liu, Jennifer Shoesmith, Alan Prescott, Luke Ramsay, Robbie Waugh, Sarah M McKim

**Affiliations:** Division of Plant Sciences, School of Life Sciences, University of Dundee, Errol road, Invergowrie, Dundee DD25DA, UK; Cell and Molecular Sciences, James Hutton Institute, Errol road, Invergowrie, Dundee DD25DA, UK; Division of Plant Sciences, School of Life Sciences, University of Dundee, Errol road, Invergowrie, Dundee DD25DA, UK; Division of Plant Sciences, School of Life Sciences, University of Dundee, Errol road, Invergowrie, Dundee DD25DA, UK; Division of Plant Sciences, School of Life Sciences, University of Dundee, Errol road, Invergowrie, Dundee DD25DA, UK; Division of Plant Sciences, School of Life Sciences, University of Dundee, Errol road, Invergowrie, Dundee DD25DA, UK; DIF and Cell Signalling and Immunology, School of Life Sciences, University of Dundee, Nethergate, Dundee DD14HN, UK; Cell and Molecular Sciences, James Hutton Institute, Errol road, Invergowrie, Dundee DD25DA, UK; Division of Plant Sciences, School of Life Sciences, University of Dundee, Errol road, Invergowrie, Dundee DD25DA, UK; Cell and Molecular Sciences, James Hutton Institute, Errol road, Invergowrie, Dundee DD25DA, UK; Division of Plant Sciences, School of Life Sciences, University of Dundee, Errol road, Invergowrie, Dundee DD25DA, UK

**Keywords:** Barley, Cuticle, Eceriferum, Grain, Pericarp, Wax

## Abstract

**The cuticle covering aerial organs of land plants is well known to protect against desiccation. Cuticles also play diverse and specialized functions, including organ separation, depending on plant and tissue. Barley shows a distinctive cuticular wax bloom enriched in** β**-diketones on leaf sheaths, stem nodes and internodes and inflorescences. Barley also develops a sticky surface on the outer pericarp layer of its grain fruit leading to strongly adhered hulls, ‘covered grain’, important for embryo protection and seed dispersal. While the transcription factor-encoding gene *HvNUDUM* (*HvNUD*) appears essential for adherent hulls, little is understood about how the pericarp cuticle changes during adhesion or whether changes in pericarp cuticles contribute to another phenotype where hulls partially shed, called ‘skinning’. To that end, we screened barley lines for hull adhesion defects, focussing on the *Eceriferum* (= waxless, *cer*) mutants. Here, we show that the *cer-xd* allele causes defective wax blooms and compromised hull adhesion, and results from a mutation removing the last 10 amino acids of the GDS(L) [**Gly, Asp, Ser, (Leu**)]-motif esterase/lipase HvGDSL1. We used severe and moderate *HvGDSL1* alleles to show that complete HvGDSL1 function is essential for leaf blade cuticular integrity, wax bloom deposition over inflorescences and leaf sheaths and pericarp cuticular ridge formation. Expression data suggest that HvGDSL1 may regulate hull adhesion independently of HvNUD. We found high conservation of HvGDSL1 among barley germplasm, so variation in *HvGDSL1* unlikely leads to grain skinning in cultivated barley. Taken together, we reveal a single locus which controls adaptive cuticular properties across different organs in barley**.

## Introduction

Land plants deposit a reflective, waxy cuticle on the outer epidermis of their aerial organs, which provides mechanical support and a physical barrier to desiccation, UV damage and other stresses. The cuticle is typically composed of a cutin polyester matrix impregnated with intracuticular waxes and overlaid with epicuticular waxes (Yeats and Rose 2013). Primarily made up of very long chain fatty acids (VLFCAs, >C20) and their derivatives, epicuticular waxes can form crystals giving the surface a glaucous (white/blue) appearance. In barley, heavy glaucous wax blooms on emerging leaf sheaths, internodes and inflorescences (spikes) consist of mostly β-diketone and hydroxy-β-diketone crystalline rods, associated with protection from UV and drought, and improved yield (Su et al. 2020). Screening for wax deficient or *eceriferum* (*cer*) mutants with glossy, green rather than glaucous appearance helped identify multiple genes involved in wax metabolism, transport and regulation in various plants (Samuels et al. 2008). Barley has a large *cer* mutant collection, several of which have been cloned, including the *CER-CQU* metabolic gene cluster responsible for β-diketone and hydroxy-β-diketone synthesis (Zhang et al. 2013, Hen-Avivi et al. 2016, Schneider et al. 2016, von Wettstein-knowles 2017) and a gene encoding a esterase/lipase characterized by a conserved GDS(L) [Gly, Asp, Ser, (Leu)] motif (Brick et al. 1995), *HvGDSL1*, involved in leaf cutin formation and water retention (Li et al. 2017).

The outer pericarp of barley grain fruit or caryopsis tightly adheres to the inner face of enclosing floral hulls, leading to *Hordeum*’s distinctive ‘covered grain’. Presence of a lipid-rich ‘cementing layer’ on the pericarp surface is thought to mediate adhesion (Harlan 1920). Poor attachment can lead to partial or total hull shed, a phenomenon called grain skinning and a major problem in barley grain end-uses, especially in malting (Bryce et al. 2010). Although we do not fully understand the causes of skinning, skinning severity varies across cultivars and responds to environmental factors, such as temperature and humidity (Brennan et al. 2017a, 2017b). While cementing layer thickness did not correlate with skinning severity across different cultivars, varieties with strong hull adhesion had a higher proportion of fatty acids and sterols and lower proportion of alkanes in their caryopsis surface lipids, suggesting a role for lipid composition in adhesion (Brennan et al. 2019). Naked barley occurs when hulls do not adhere to the caryopses and shed free. Selected early in barley domestication for human consumption, all naked cultivars derive from the deletion of the *NUDUM* gene (*HvNUD*) encoding an ETHYLENE RESPONSE FACTOR (ERF)/APETALA-2 (AP2) transcription factor homologous to the *Arabidopsis* cuticular regulator WAX-INDUCER-1/SHINE-1 (Aharoni et al. 2004, Taketa et al. 2008). Gaines and colleagues showed that naked barleys lacked cuticular thickenings thought to represent the cementing layer (Gaines et al. 1985). Naked barley pericarps did not stain with Sudan Black B (SB), a lipid-reactive dark dye, in contrast to covered barley, and so SB staining was suggested to reflect cementing layer deposition (Taketa et al. 2008). Interestingly, *HvNUD* expression was restricted to the testa or seed coat layer under the pericarp. We know little about the steps between *HvNUD* expression and hull adhesion; however, three allelic barley *cer* mutants, *cer-yl, cer-ym* and *cer-zv*, reportedly generate naked grain (Lundqvist and Franckowiak 1996, Taketa et al. 2008). We hypothesized that other genes involved in cuticular wax production may also contribute to hull adhesion and potentially to grain skinning. To explore this hypothesis, we screened all barley *cer* mutants within the Bowman Near Isogenic Lines (BWNILs), a large collection of mutants introgressed into the background cultivar Bowman (Druka et al. 2011), and identified mutants showing grain skinning. In this paper, we show that the *cer-xd* locus encodes a *HvGDSL1* variant which causes poor hull to caryopsis attachment and changes the pericarp cuticle.

## Results

### The *cer-xd* allele is a mutation in the SGNH family esterase/lipase encoding gene *HvGDSL1*

**Fig. 1:**
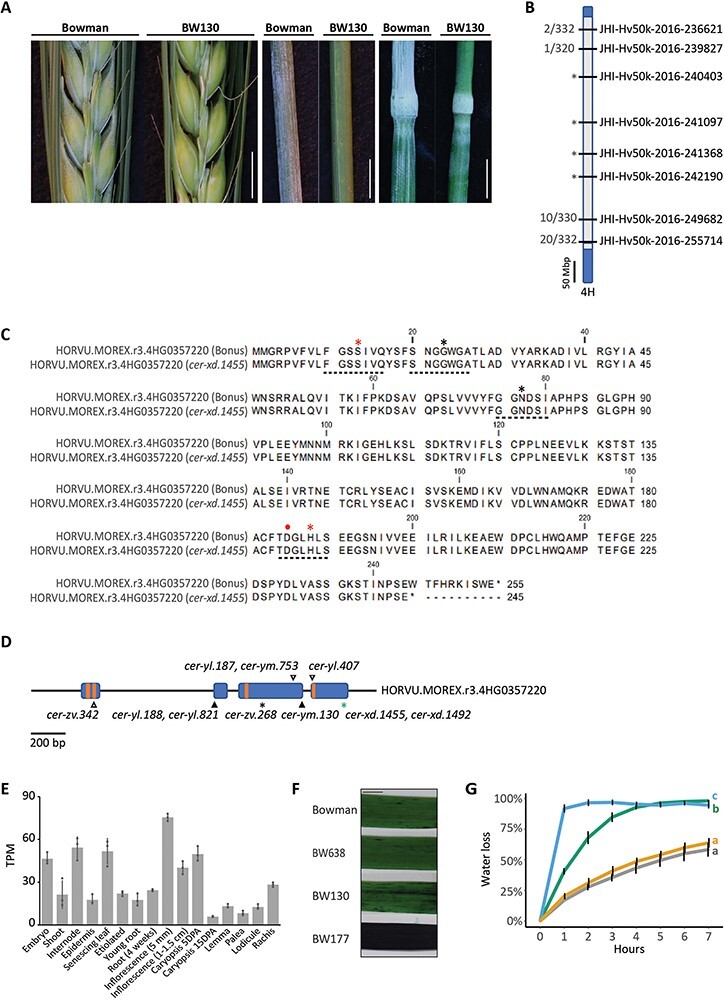
Variation in a *GDSL* esterase/lipase underlies the *cer-xd* locus in barley. (A) Wax bloom deposition on spikes, leaf sheaths and stems (nodes and internodes) of Bowman and BW130. Scale bars = 5 mm. (B) Representation of chromosome 4H in BW130 showing an introgression from *cer-xd.1455* (gray) into Bowman (blue). Labels on right indicate the eight markers on 4H run on the mapping population; labels on left indicate proportion of recombinant gametes. Asterisks indicate markers in complete linkage with the glossy phenotype. (**C**) Protein alignment of HORVU.MOREX.r3.4HG0357220 in Bonus and *cer-xd.1455*, showing the premature stop codon removing the last 10 amino acids in the latter. Dotted lines underneath indicate the four *GDSL* conserved blocks, asterisks indicate the SGNH conserved amino acids, while red asterisks and dot indicate amino acids that form the catalytic triad. (D) HORVU.MOREX.r3.4HG0357220 gene model. Blue boxes represent exons and orange boxes the four *GDSL* conserved blocks. Variants of *cer-yl, cer-ym* and *cer-zv* identified in (Li et al. 2017) are shown in black and the *cer-xd* variant identified in this manuscript is shown in green. Triangles indicate amino acid substitutions and asterisks indicate premature stop codons. (E) Expression of HORVU.MOREX.r3.4HG0357220 in Morex tissues from the Barley Expression Database (Milne et al. 2021). Data expressed in transcripts per million (TPM). Bars represent mean expression with standard deviation, dots represent biological replicates. (F) Representative images of TB staining of the 2nd leaf of 3-week-old plants. Scale bar = 10 mm. (G) Percentage water loss from the 2nd leaf of 2-week old plants. Lines show average and error bars show standard deviation. Line color indicates genotype: gray, Bowman; green, BW130; blue, BW177; and yellow, BW638. Letters indicate significant differences of the areas under the curves (*P* = 0.05; Tukey’s HSD multiple comparison following one-way ANOVA), *N* = 4.

Hand-threshing revealed decreased hull adhesion in the BWNIL BW130. BW130 derives from a cross between Bowman and the sodium azide-generated recessive mutant *cer-xd.1455* (in *cv*. Bonus) described to have reduced spike, leaf sheath and stem wax (Lundqvist and Lundqvist 1988). BW130 shows an additional phenotype of extra surface wax on stem nodes (Druka et al. 2011). Genotyping BW130, Bowman and Bonus with the 50k iSelect genotyping *SNP* array (Bayer et al. 2017) revealed multiple donor introgressions on six of barley’s seven chromosomes (**Supplementary Tables S1, S2**). To map the *cer-xd* locus, we grew a Bowman × BW130 BCF_2_ population, phenotyped for spike and leaf sheath glossiness and genotyped with 22 *KASP* (LGC Biosearch Technologies, Teddington, UK) markers encompassing all BW130 introgressions (**Supplementary Table S3**). We observed the glossy phenotype in 40 out of 168 F_2_ plants, confirming 1:3 segregation (χ^2^ = 0.127; *P*  = 0.716). We found complete linkage between *cer-xd* and four markers in the centromeric region of chromosome 4 H (H for *Hordeum*) (JHI-Hv50K-2016-240403; JHI-Hv50K-2016-241097; JHI-Hv50K-2016-241368; JHI-Hv50K-2016-242190; **Fig. 1B**). *HvGDSL1* (HORVU.MOREX.r3.4 HG0357220) is positioned on 4 H distal to the JHI-Hv50k-2016-240403 marker. Loss of function variation in this gene underlies three allelic barley *cer* mutants, *cer-ym, cer-yl* and *cer-zv*, showing glossy spikes and leaf sheaths, wax covered nodes, increased leaf cuticle permeability and naked seeds (Li et al. 2013, 2015, 2017). *GDSL*-motif esterase/lipases are characterized by four or five highly conserved amino acid sequences (blocks) with the GDS(L) motif in Block I, close to the N terminus of the protein; the Ser in block I together with Gly, Asn, His, in block II, III and IV, respectively, are required for catalytic activity and are always present in these enzymes, also known as SGNH lipases (Akoh et al. 2004, Ding et al. 2019); **Fig. 1C, D**). Sequencing *HvGDSL1* in BW130, the original *cer-xd.1455* and *cer-xd.1492* (an additional *cer-xd* allele in Bonus) revealed that they all shared an identical fourth exon *SNP*, absent in Bowman and Bonus, causing a predicted premature stop codon (TGG to TGA, W^245^ to stop) removing the last 10 amino acids of *HvGDSL1* (**Fig. 1C, D**). *HvGDSL1* has wide expression with enrichment in reproductive tissues such as inflorescences and developing caryopses, as well as internodes [**Fig. 1E**; Barley Expression Database (Milne et al. 2021)], consistent with *cer-xd* phenotypes. We suggest that *cer-xd* is an allele of *HvGDSL1*.

To compare *cer-xd* with the other *HvGDSL1* alleles, we selected BW177, a BWNIL which harbors *cer-zv.268*. Sequencing BW177 confirmed the A to T *SNP* shown in Li et al. (2017) that causes an early premature stop codon (AAG to TAG, K^108^ to stop). Similar to *cer-ym, cer-yl* and *cer-zv*, BW177 had no wax bloom over the spike and leaf sheaths (**Supplementary Fig. S1A**), compared to the intermediate phenotypes observed in BW130. Li et al. (2013) showed that *cer-zv* has more permeable leaves associated with a decrease in leaf cutin monomers (Li et al. 2013). Therefore, we tested leaf cuticle permeability in BW130, BW177 and Bowman leaf blade fragments using Toluidine Blue (TB) penetration and water loss assays. After 2 h immersion in TB solution, Bowman showed no uptake of TB, while BW130 leaf fragments were partially stained and BW177 leaf fragments were saturated (**Fig. 1F**). BW130 and BW177 detached leaves dried more quickly than Bowman over time (*P* < 0.001), losing 40 and 92% total water content, respectively, after 1 h at 30⁰C, compared to only 17% lost in Bowman (**Fig. 1G**). BW177 developed slightly smaller stomata but equivalent stomatal density to Bowman suggesting that differences in water loss between these genotypes mostly reflect differences in cuticle permeability (**Supplementary Fig. S2**). Altogether, *cer-xd* leaves, leaf sheaths and hull phenotypes were intermediate between wild-type and *cer-zv*, suggesting some *HvGDSL1* function in *cer-xd*.

*HvGDSL1* retains the secondary structure of other known α/β hydrolases based on both amino acid sequence [alphafold; UniProt: F2DIQ7; (Jumper et al. 2021, Varadi et al. 2022)] and homology to protein with a known crystal structure [Phyre2; model based on *S.cerevisiae* c3milA; (Kelley et al. 2015)]: four to five parallel β-strands surrounded by seven to eleven α-helices forming a relatively rigid core structure around the central binding pocket (Akoh et al. 2004, Anderson et al. 2022) (**Supplementary Fig. S3A, B**). While the C-terminal 10 amino acids lie outside these structures, the *cer-xd* phenotypes suggest that this deleted region reduces *HvGDSL1* function (**Supplementary Fig. S3C**). *HvGDSL1* clusters in a subfamily distantly related to other *GDSL* lipase gene family members (Park et al. 2010, Li et al. 2017), whose orthologues show high identity across monocots (average identity of the protein sequence 85%) and dicots (average identity of the protein sequence 68%). Interestingly, the last 10 amino acids show high conservation across all tracheophyte orthologs examined. Other barley *GDSL* sequences show much lower sequence identity; even the closest paralogs only have a sequence identity of 39% and lack conservation of the last 10 amino acids (**Supplementary Fig. S3D, E**; **Supplementary Table S4**). The *cer-xd* phenotypes and conservation of the 10 amino acids across orthologs suggest an important role for the final 10 amino acids in leaf cuticle integrity and deposition of the wax bloom over spike and leaf sheath in barley and potentially important roles in orthologs.

### Final 10 amino acids in the *HvGDSL1* C-terminus contribute to hull to caryopsis attachment

**Fig. 2:**
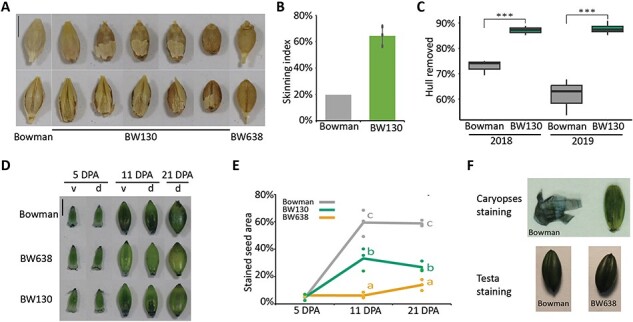
Grain features of BW130. (A) Grain skinning in BW130 compared to Bowman (covered grain) and BW638 (*nud*, naked grain). Scale bar = 5 mm. (B) Skinning index (percentage of seed showing skinning). Bars indicate the average value with standard deviation of three biological replicates (dots) from BW130 or one replicate representing seed from three pooled individuals from Bowman. Over 150 grains inspected per replicate. (C) Box plots show hull removed as percentage of total hull weight in Bowman and BW130 after 15 s of debranning. The lower and upper box represent the first and the third quartiles, the horizontal line indicate the median, the lower and upper whiskers represent the minimal and maximal values within 1.5 times interquartile range. Significant differences following one-way ANOVA (*** = *P* < 0.001), *N* ≥ 3. (D) SB staining of Bowman, BW638 and BW130 caryopses; v, ventral and d, dorsal. Scale bar = 5 mm. (E) Percentage of dorsal seed surface area stained by SB, lines represent average and dots represent individual bioreplicates (each bioreplicate is the average of two grains from the same spike). Letters indicate significant differences (*P* < 0.05; Tukey’s HSD multiple comparison following one-way ANOVA), *N* = 3, except for 5 DPA BW130, where *N* = 2. (**F**) Top panel shows tissue dissection of 11 DPA caryopses of Bowman after staining with SB. Tissue on left is the pericarp dissected off the caryopsis shown on the right. Removal of the pericarp reveals the testa on the caryopsis which is also stained. Bottom panel shows 11 DPA caryopses of Bowman and BW638, where the pericarp was removed to reveal the testa before staining with SB.

Grain from the *HvGDSL1* mutant alleles, *cer-yl, cer-ym* and *cer-zv*, are reportedly naked (Lundqvist and Franckowiak 1996). We found that BW130 grains showed a range of hull loss compared to Bowman, from naked to covered (**Fig. 2A**), with an average of 64%, compared to only 20% grain skinning in Bowman (**Fig. 2B**). To quantify attachment strength, we developed a skinning assay comparing our reference Bowman to Concerto, a cultivar strongly susceptible to skinning (Brennan et al. 2017b) using a small-scale debranner (Satake) that removes the hull by progressive abrasive action. Testing different sample weights and debranning time we determined that a sample weight of 25 g and a debranning time of 15 s was optimal to differentiate these genotypes (**Supplementary Fig. S4**). Using this assay on grain harvested in two seasons (2018 and 2019) showed that BW130 grain lost 87% (2018) and 88% (2019) of the total hull by weight, significantly higher to the 73% (2018) and 61% (2019) lost in Bowman (*P* < 0.001) (**Fig. 2C**; **Supplementary Table S5**) confirming weaker hull to caryopsis attachment in BW130 compared to Bowman. Taken together, variation in *HvGDSL1* in *cer-xd* leads to grain skinning.

We next asked whether *HvGDSL1* regulates pericarp surface features by examining pericarps of developing grain of BW130, Bowman and a naked control, the BW638 line carrying the *Hvnud* deletion, selecting three developmental stages of 5, 11 and 21 days post-anthesis (DPA). Adhesion differed across genotypes and stages. BW638 hulls never stick to the pericarp. Hulls from 5 and 11 DPA Bowman and BW130 grains were easily removed, although 11 DPA Bowman caryopses were stickier to touch compared to BW130; by 21 DPA, Bowman and BW130 hulls adhered; however, dorsal hulls could be removed whole while ventral hulls could only be removed whole in BW130 and usually fractured on removal in Bowman (**Supplementary Table S6**). We tested for surface lipids by staining these caryopses with SB. SB stained at the apical ends of caryopses at 5 DPA for each genotype, ranging from 4% to 6% (**Fig. 2D, E**). BW638 caryopses showed similar apical end staining at 11 DPA (6%) and 21 DPA (14%; BW177 caryopses, also naked, remained unstained by SB at 18 DPA, **Supplementary Fig. S1A**). At 11 DPA, SB stained 59% of the dorsal side of Bowman caryopses but a smaller fraction, 33%, of BW130 dorsal area (*P* < 0.001). By 21 DPA, 59% of the dorsal side of Bowman caryopses stained faintly and only 27% of the dorsal side of BW130 even more lightly stained (*P* < 0.001). Thus, SB staining of BW130 appeared intermediate between Bowman and naked lines (BW638 and BW177). Interestingly, removing the pericarp following SB staining of 11 DPA Bowman caryopses revealed a very darkly stained testa (seed coat) (**Fig. 2F**). Brennan et al. (2017a) reported pericarp damage following removal of adherent hulls, which we also observed (**Supplementary Fig. S5**). We speculated that pericarp damage from removing adhering hulls from 11 DPA onwards allows the stain to penetrate through the testa. Although Taketa et al. (2008) explained intense staining of Bowman caryopses from 14 and 21 DPA as due to cementing layer accumulation on the pericarp (Taketa et al. 2008), we suggest that this could be a combination of pericarp and testa staining. Rather than lack of surface lipids or cementing layer, absence of dark staining in BW638, as well as BW177, may instead represent an intact pericarp as non-adherent hull removal does not cause pericarp rupture (removal of the pericarp from naked grain revealed that the testa stains readily with SB, **Fig. 2F**; SB pericarp and testa staining were also observed in caryopses cross-section by Taketa et al. 2008). Thus, variation in SB staining could reflect different levels of hull attachment strength, and consequently pericarp damage following hull removal; however, it is also possible that pericarp cuticles in adherent lines become more permeable independently from hull attachment, which could lead to SB penetrating to the testa. Altogether, our data support compromised adhesion in BW130, correlating with fainter SB staining observed BW130 caryopsis compared to Bowman (**Fig. 2D, E**).

### Variation in *HvGDSL1* influences pericarp cuticle thickness and surface features

**Fig. 3:**
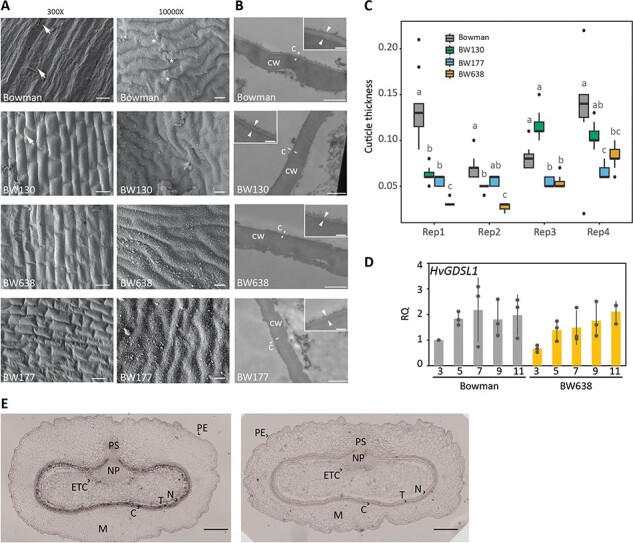
Cuticular features of the grain defective mutants. (A) Scanning electron micrographs of the pericarp surface of 11  DPA caryopses at 300× (scale bars = 50 µm) and 10,000× (scale bars = 1 µm) magnification (hulls removed). Arrows show broken pericarp and asterisks indicate accumulating material on cuticular ridges. (B) Transmission electron micrographs of a cross section of a central segment (3 mm long × 1 mm wide × 1 mm thick) of Bowman, BW130, BW177 and BW638 caryopses at 11 DPA showing the pericarp cuticle. C = cuticle, CW = cell wall. Arrows indicate cuticle. Scale bars = 1 µm; inlets scale bars = 150 nm. (C) Box plots representing the pericarp cuticle thickness (µm) of 11 DPA caryopsis. Four biological replicates plotted separately along the *x*-axes. Each biological replicate comprises 11 measurements on independent images (except Bowman Rep1 which represents seven measurements). The lower and upper box represent the first and the third quartiles, the horizontal line indicate the median, the lower and upper whiskers represent the minimal and maximal values within 1.5 times interquartile range, dots represent outliers beyond this range. Letters indicate significant differences between genotypes within each replicate (*P* < 0.05; Dunn’s test following Kruskal–Wallis test). (D) qRT-PCR of *HvGDSL1* transcript levels in Bowman and BW638 (*nud*) at 3, 5, 7, 9 and 11 DPA. Dots indicate individual biological replicates and bars indicate average relative expression with standard deviation. RQ = relative quantification, calculated relative to one Bowman replicate. (E) In situ hybridization of 5 DPA caryopses sections with *HvGDSL1* antisense (left) and sense (right) probe. Scale bars = 200 µm. PE = pericarp epidermis, M = mesocarp, C = chlorenchyma, T = testa, N = nucellus, ETC = endosperm transfer cells, NP = nucellar projection, PS = pigment strand.

To better understand pericarp features controlled by *HvGDSL1*, we examined Bowman, BW130, BW177 and BW638 pericarps at higher resolution. Scanning electron microscopy (SEM) of 11 DPA Bowman caryopses showed cuticular deposition on elevated ridges of the pericarp (**Fig. 3A**, asterisks), potentially cementing layer, and surface tears revealing reticulated cell walls of the testa (**Fig. 3A**, arrows; **Supplementary Fig. S5**). BW130 surfaces appeared smoother with less ridge deposition, while BW638 and BW177 showed smooth, undamaged surfaces, and few thickened ridges (**Fig. 3A**). Examining pericarp cuticle ultrastructure with transmission electron microscopy (TEM) showed variability within the replicates; however, BW638 shows cuticular layers between 0.2 and 0.7 times thinner compared to Bowman. BW130 and BW177 showed intermediate, variable cuticular layers between 0.4 and 0.7 times thinner compared to Bowman, with one replicate (BW177) and two (BW130) being not significantly different (**Fig. 3B, C**). *HvGDSL1* transcripts detected in Bowman caryopses increased in level after 3 DPA and remained consistent during hull adhesion (**Fig. 3D**). *In situ* hybridization of 5 DPA grain with antisense *HvGDSL1* probe showed signal in the inner integuments which will form the testa, the endosperm transfer cells and very weak signal in the pericarp epidermis (**Fig. 3E**). Taken together, *HvGDSL1* promotes thickened cuticles associated with the presence of cuticular ridges and strong hull adhesion. These roles appear concurrent with *HvGDSL1* expression, although the strong expression in the testa suggests that *HvGDSL1* may perform additional functions in that tissue which also develops a prominent cuticle.

### Extractable caryopses surface lipids change during hull adhesion

**Fig. 4:**
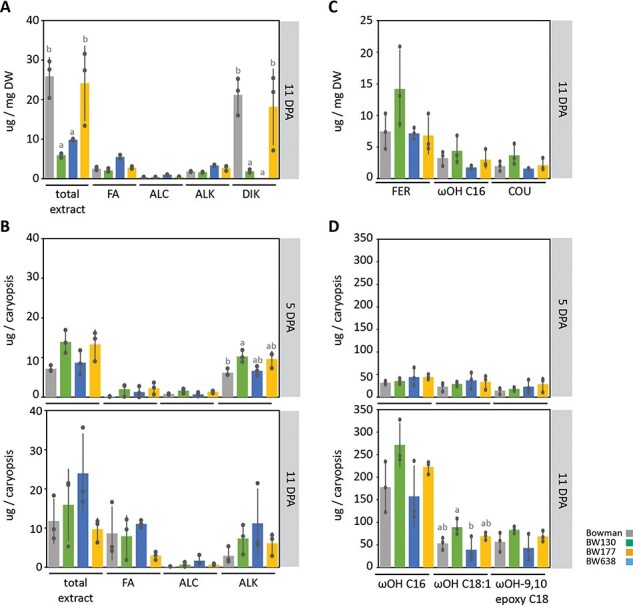
Surface lipids on hulls and caryopses during adhesion. Profile of extracted surface lipids from hulls (A) and caryopses (B) and cutin monomers from hulls (C) and caryopses (D) showing the average (bars) of independent bioreplicates (dots) with standard deviation. For the surface lipid extracts, total extract and sum of the major wax species are shown (FA = fatty acids, ALC = alcohols, ALK = alkanes, DIK = diketones). For cutin extracts major components are shown (COU = coumaric acid methyl ester, TMS; FER = ferulic acid methyl ester, TMS; ωOH C16 = ω-hydroxyhexadecanoic acid; ωOH C18:1 = ω-hydroxyoctadecenoic acid; ωOH-9,10 epoxy C18 = ω-hydroxy-9,10 epoxy octadecanoic acid). Letters indicate significant differences within genotypes (*P* < 0.05; Tukey’s HSD multiple comparison following one-way ANOVA). DW = hull dry weight. Side panels indicate sample stage. N = 3 except for BW177 and BW638 surface lipid extracts at 5 DPA where N = 2.

Since mutations in *HvGDSL1* reduce visible spike and leaf sheath wax and cause defective leaf cuticle formation (our work and Li et al. 2017), we profiled extractable surface lipids and cutin monomers from hulls (11 DPA) and caryopses (5 and 11 DPA). The β-diketones and OH-β-diketones responsible for the wax bloom dominated the Bowman hull lipid profile and together made up 82% of the total extracted lipids. BW638 hulls had amounts of β-diketones and OH-β-diketones (80% of total extract) equivalent to Bowman (**Fig. 4A**), consistent with no reported difference in leaf sheath or spike visible wax (**Supplementary Fig. S1B**). The amount of surface lipids extracted from BW130 and BW177 hulls was 76% and 71% lower, respectively, compared to Bowman (**Fig. 4A**, *P* < 0.001). Correlating with their wax phenotypes (**Fig. 1A**; **Supplementary Fig. S1A**), β-diketones and OH-β-diketones were under the detection limit in BW177 and accounted for only 31% of the total extract in BW130 (**Fig. 4A**). Fatty acids, alcohols and alkanes represented minor proportions of 10%, 1.4% and 7.1%, respectively, in Bowman extracted lipids and accumulated to comparatively lower amounts in BW130 and BW177 for the longer chain fatty acids (C28 to C32), alcohols (C30-C32) and alkanes (C31) (**Supplementary Table S7**). Taken together, *HvGDSL1* function promotes accumulation of β-diketones, OH-β-diketones and longer chain (> C28) aliphatic compounds in barley hulls, consistent with *cer-xd* and *cer-zv* wax bloom defects.

Alkanes (86%), alcohols (11%) and fatty acids (2.2%) were the main components of Bowman total extracted surface lipids from 5 DPA caryopses (**Fig. 4B**). At 5 DPA, caryopses surface lipids showed a higher proportion of alkanes in BW130 (C23, C25), BW177 (C23) and BW638 (C23, C25), while the other components were equivalent to Bowman (**Fig. 4B**; **Supplementary Table S8**). Caryopsis surface lipids changed markedly in Bowman between 5 and 11 DPA. The composition became dominated by fatty acids (73%), followed by alkanes (25%) and alcohols (1.6%) with fatty acids (C14, C18, C26) increasing and both alcohols (C24 to C28) and alkanes (C23) decreasing. BW177 also showed an increased proportion of fatty acids and alcohols, while BW130 and BW638 both decreased the proportion of alcohols and alkanes. Potentially, due to high variability of replicate samples, no differences among genotypes in the surface lipid composition were significant at 11 DPA (**Fig. 4B**; **Supplementary Table S8**). Taken together, fatty acids significantly increase on the caryopsis surface concomitant with hull adhesion, particularly of short chains (C14–C18), balanced by a reduction in alkanes and alcohols. However, variation in *GDSL1* or NUD function did not correlate with caryopses lipid changes which could explain differences in hull attachment.

We also extracted cutin monomers from hulls and caryopses cuticles. Barley leaf cutin has variable proportions of mono and di-hydroxy C16 and C18 acids (Richardson et al. 2007, Chen et al. 2011, Li et al. 2013, 2015, 2018). In Bowman hulls, we found the most abundant component were aromatics, in particular ferulic and coumaric acid, followed by ωOH C16, while only a minor proportion of ωOH 9,10 epoxy C18 is present (**Fig. 4C**, **Supplementary Table S9**). BW130, BW177 and BW638 showed a similar composition with variation in amounts not statistically significant (**Fig. 4C**, **Supplementary Table S9**). In contrast, the cutin monomer composition from caryopses extract showed the typical mono and di-hydroxy C16 and C18 acids present in leaves, however with a higher proportion of the C16 moiety. Between 5 and 11 DPA all monomers increased (between 2.3- and 10-fold). The other lines analyzed showed a similar composition and increasing trend without statistically significant differences (**Fig. 4D**; **Supplementary Table S10**). These data show that the cutin composition profile of the caryopsis is distinct from hull and from leaf cutin profiles. Total amounts of cutin increased concomitant with hull adhesion, yet showed no differences across the lines suggesting that different levels of extractable cutin do not correlate with differences in hull adhesion.

### Genetic network of hull adhesion control

**Fig. 5:**
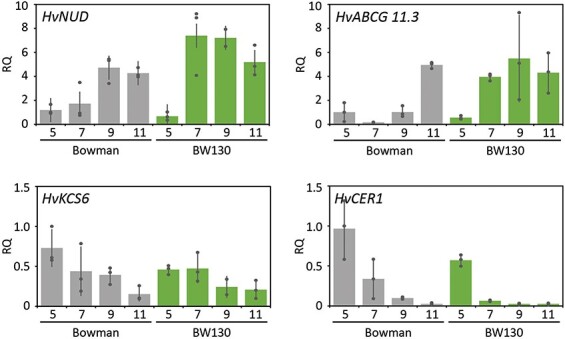
Relative expression of cuticle-associated genes. qRT-PCR shows relative expression of genes associated with adhesion and cuticle metabolism in caryopses of Bowman and BW130 at 5, 7, 9, and 11  DPA. Dots indicate individual biological replicates and bars indicate average relative expression with standard deviation, *N* = 3. RQ = relative quantification, calculated relative to one Bowman replicate.

Severe loss of *HvGDSL1* function alleles causes naked grain and the more moderate *cer-xd* allele causes skinning, demonstrating that the extent of *HvGDSL1* controls the strength of hull adhesion. Since WIN1/SHN-like transcription factors have been associated with cuticular gene regulation (Kannangara et al. 2007), and both HvNUD and *HvGDSL1* control hull adhesion, we hypothesized that *HvGDSL1* could be a downstream target of HvNUD. We detected no differences in *HvGDSL1* expression comparing BW638 and Bowman developing grain at 3, 5, 7, 9 and 11 DPA by qRT-PCR, indicating that HvNUD is not required for *HvGDSL1* expression (**Fig. 3D**). We found increased expression of *HvNUD* in BW130 caryopses, in particular at 7 DPA (4.3-fold increase), as well as increased levels at 7 and 9 DPA (24- and 5.4-fold increase, respectively) of *HvABCG11.3*, a homolog of the *Arabidopsis* (*At*) *ABCG11*, encoding a transporter responsible for trafficking cuticle and wax precursors across the plasma membrane (**Fig. 5**; Bird et al. 2007, Panikashvili et al. 2011). *HvKCS6*, involved in the elongation of fatty acids with chain length higher than C22 (Weidenbach et al. 2014), and *HvCER1*, homolog of *AtCER1* responsible for the synthesis of alkane from VLCFA (Bourdenx et al. 2011), were both downregulated at 5 DPA (*HvKCS6* and *HvCER1*, 0.63- and 0.59-fold decrease, respectively) and at 9 DPA (*HvCER1*, 0.25-fold decrease), which may contribute to reduced longer chain fatty acids (C28–C32) and alkanes (C31) in BW130 hulls (**Fig. 5**; **Supplementary Table S7**). Altogether then, HvNUD does not regulate *HvGDSL1* expression, while functional *HvGDSL1* appears necessary for normal expression of *HvNUD* and other genes involved in cuticular biosynthesis and transport.

### *HvGDSL1* appears highly conserved in the barley germplasm

**Fig. 6:**
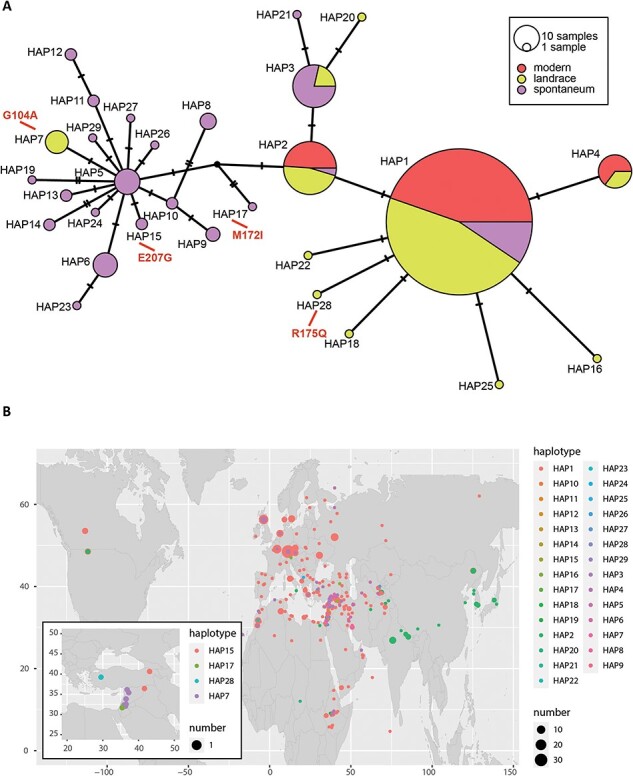
Haplotype analysis of HORVU.MOREX.r3.4HG0357220. (A) Median-joining network for HORVU.MOREX.r3.4HG0357220 HAPs. Single-nucleotide polymorphisms (*SNPs*) were identified comparing HORVU.MOREX.r3.4HG0357220 exonic regions of 477 diverse *Hordeum spontaneum* (wild barley) and *Hordeum vulgare* (modern and landraces) accessions. Node size is relative to HAP frequency. Bars between two nodes indicate the number of nucleotides within the sequence that differ between haplotypes. Amino acid changes are shown in red. (B) Haplotype geographical origin distribution. HAPs are distinguished by colors and sizes as described in the legend. Panel inset shows distribution of HAPs with amino acid changes.

Grain skinning is observed across multiple barley cultivars (Brennan et al. 2017b). To determine whether *HvGDSL1* variants could contribute to skinning in barley germplasm, we assessed *HvGDSL1* sequence variation in exome capture sequence data from 477 wild (*H. spontaneum*) and cultivated (*H. vulgare*) barley accessions (Russell et al. 2016, Bustos‐Korts et al. 2019; **Supplementary Table S11**). We identified 32 variations across the coding sequence, 11 at exonic sites: seven in exon III, 2 in exon IV and one each in exon I and exon II. Of the exonic variants, four caused non-synonymous changes (G104A, M172I, R175Q in exon III and E207G in exon IV). All non-synonymous changes fall outside the four conserved blocks defining the SGNH motif of the *GDSL* lipases (**Supplementary Table S12**). Variants arranged into 29 haplotypes (HAP, **Fig. 6A**), with HAP1 alone containing 329 lines (69% of total). HAPs 1–3 together contained 400 (out of 477, 84%) lines and were the only HAPs represented in both wild and cultivated (including landraces) accessions. Modern cultivated accessions are only represented in HAPs 1, 2 and 4, which differed from each other by a single synonymous *SNP*, indicating *HvGDSL1* varies little across the cultivated germplasm (**Supplementary Table S11**). Non-synonymous mutations were found exclusively outside of modern cultivars, with HAP7 (G104A) present in eight landraces, HAP15 (E207G) composed of two wild accessions, HAP17 (M172I) in a single wild accession and HAP28 (R175Q) in one landrace. These HAPs were limited to regions of early barley domestication, indicating that none of these alleles were adopted during barley’s spread across its current range of expansion (**Fig. 6B**). In contrast, HAPs 4, 7, 16, 18, 20, 22, 25 and 28 represent exclusively cultivated accessions (including landraces), suggesting that these variants emerged after barley domestication. Haplotype analysis suggests high conservation of *HvGDSL1* in cultivated germplasm, so *HvGDSL1* variation unlikely contributes to skinning in modern cultivars.

## Discussion

We show that the *cer-xd* mutant is caused by an early stop codon removing the last 10 amino acids in *HvGDSL1*. The *cer-xd* phenotypes of reduced wax bloom, increased leaf cuticle permeability and grain skinning are intermediate compared to the highly permeable leaf cuticles, glossy leaf sheath and spikes and naked caryopses previously described for allelic mutants, *cer-yl, cer-ym* and *cer-zv*. We suggest that these phenotypic differences arise due to differences in *HvGDSL1* function with the severe allele amino acid substitutions in the highly conserved alpha folds and β-sheet domains of *HvGDSL1* (Li et al. 2017), causing a more substantial loss of *HvGDSL1* function compared to the deletion of the 10 amino acids at the C-terminal region in *cer-xd*. Nonetheless, the *cer-xd* intermediate phenotypes demonstrate that these amino acids, while outside of the known functional domains, are essential for complete *HvGDSL1* function to promote wax blooms, cuticle integrity and strong adhesion to the caryopsis, revealing new insight about *HvGDSL1* C-terminal residues. Furthermore, conservation of these amino acids across orthologs in monocots, dicots and tracheophytes, suggests functional relevance across vascular land plants, for instance by impacting substrate specificity, stability or alignment as in other C-termini of SGNH *GDSLs* (Anderson et al. 2022).

*GDSL* esterases/lipases are found across organisms, with particularly high numbers and diverse substrates in plants, although most remain uncharacterized (reviewed in Shen et al. 2022). While plant *GDSLs* play roles in epidermal integrity and surface specializations, their control of cuticular composition depends on the species and/or tissues. Previous work showed that *HvGDSL1* promotes cutin synthesis in leaves but not the accumulation of wax or of the main wax leaf component, C26 alcohol (Li et al. 2013; Li et al. 2015). We demonstrate that hull cutin components are not affected by *HvGDSL1*, but that *HvGDSL1* function is important for accumulation of β-diketones and OH-β-diketones, as well as fatty acids (C28–C32) and alkanes (C31) in hulls (**Fig. 1**), extending the biological role of *HvGDSL1* to inflorescence organs. Altogether, we reveal that *HvGDSL1* has tissue-specific roles within barley and is required for full wax blooms.

The barley pericarp grows mostly by cell expansion as the grain fills sufficiently to contact the encasing hulls (Radchuk et al. 2011). *HvGDSL1* expression in the grain increases before and remains level throughout adhesion, and appears enriched in the testa and pericarp (**Fig. 3D, E**). Investigating pericarp development more closely revealed thickened pericarp cuticles and deposition of material over ridges in Bowman, features almost absent in BW177 or BW638, which also have thinner pericarp cuticles, suggesting a novel role for *HvGDSL1* in promoting surface morphology changes and thickness during early caryopsis development, coincident with increased expression of *HvGDSL1* and hull to caryopsis adhesion (**Fig. 2**). *GDSLs* in dicots also control surface features such as pericarp thickness and cuticular ridges formation. The extracellular *GDSL* lipase CUTIN SYNTHASE-1 (CUS1) promotes cuticle deposition in the outer pericarp of tomato fruit during cell expansion (Segado et al. 2020), while downregulation of the tomato *GDSL1* results in thinner pericarp associated with decreased cutin and epicuticular wax (Girard et al. 2012). The *Arabidopsis* ortholog of CUS1, CUTIN SYNTHASE-2 (AtCUS2), maintains cuticular ridges on sepals potentially by enabling continual cuticle deposition as sepal epidermal cells expand during maturation (Hong et al. 2017). Thus, similar to AtCUS2, *HvGDSL1* may help maintain continuous cuticle deposition in pace with caryopsis growth, which could promote adhesion and ridges. In contrast to hull wax, we did not detect major differences in extracted pericarp lipids or cutin in either *cer-xd* or *cer-zv* compared to wild-type, despite impaired hull to pericarp adhesion and thinner and less reticulated cuticles. Other studies similarly observed that *GDSLs* can cause cuticular defects without marked changes in extracted wax and/or cutin. For example, loss of function of the rice ortholog Wilted Dwarf Lethal-1 (WDL1) caused high cuticle permeability, disorganized epicuticular wax crystals and uneven cuticle thickness—similar phenotypes to defective function of *HvGDSL1*—yet no changes in composition or amount of cuticular wax or cutin monomers (Park et al. 2010), while the tomato *cus1* mutant had no effect on cutinosome number yet exhibited later changes in cuticle thickness (Segado et al. 2020). We speculate that although we did not detect major changes in cuticle and wax accumulation over the pericarp in the *HvGDSL1* mutant alleles, minor changes could affect lipid crystallization which may affect its adhesive properties.

While defective *HvGDSL1* alleles show both glossy hulls and weakened hull adhesion, we do not propose a mechanistic link between glossy hulls and defective adhesion since many other *cer* BWNILs with glossy spikes do not show defects in hull attachment (data not shown). Furthermore, most wild barleys have glossy spikes and adherent hulls (Nice et al. 2017). In fact, defective cuticles and impaired cuticular integrity are more commonly described as causing organ adhesion, not preventing it: for example, thinner yet glossier cuticles in the *sticky peel* (*pe*) mutant of tomato are associated with stickiness (Nadakuduti et al. 2012), while *Arabidopsis* and maize cuticle mutants show organ fusion phenotypes due to cuticular adhesion during development (Jenks et al. 1996, Rowland et al. 2007, Liu et al. 2021). Our initial hypothesis was that *cer-xd*, which we now know is *HvGDSL1*, could represent a target downstream of HvNUD important to modulate cuticular deposition necessary for hull adhesion, thus mechanistically linking the gene variant responsible for naked grain with genetic variation leading to skinning. However, we show that *HvGDSL1* transcript levels are not regulated by HvNUD. Other genes and mechanisms could be important to regulate *HvGDSL1* and contribute to the genetic basis for partial hull shed. *HvGDSL1* promotes expression of the genes encoding HvKCS6, HvABCG11.3 and HvNUD. We hypothesize that one of the products resulting from HvGDSL1 enzymatic activity may act as a feedback regulator of the lipid synthesis and transport pathways.

We discovered a shared genetic basis for wax blooms deposition over the hull cuticle and hull adhesion to the pericarp cuticle, two distinctive adaptive features in barley. The adherent hull may help protect the embryo and retain the attached awn important for seed dispersal while the hull protects grain during storage as well as acts as a filter during the malting process (Harlan 1968, Su et al. 2020). Conversely, due to the ease of food preparation, naked barley became dominant for human consumption across Europe during the bronze age and persists as an important calorie source, especially in high altitude regions of Asia and Ethiopia (Newman and Newman 2006). Wax blooms are associated with increased abiotic stress tolerance, protecting vegetative tissues exposed to increased light and potential stresses while elongating during the reproductive phase (Su et al. 2020). Perhaps due to the importance of both traits to barley growth and successful seed production, our data show a high conservation of *HvGDSL1* across the cultivated germplasm. Intriguingly the more extreme mutations of *HvGDSL1* (*cer-ym, cer-yl* and *cer-zv*) are described as semidwarf, with delayed heading and high level of sterility (Lundqvist and Franckowiak 1996). The high conservation identified makes it unlikely that variation in *HvGDSL1* explains skinning in cultivated varieties. However, *HvGDSL1* could be a target for factors controlling skinning in the field, which could modulate *HvGDSL1* expression or function. In summary, we show that a variant of a *GDSL*-motif esterase/lipase causes poor hull to caryopsis attachment in barley, which could represent a NUD-independent route to changes in hull adhesion.

## Material and Methods

### Plant material

BW130, BW177 and BW638 are barley near isogenic lines (NIL) derived by crossing respectively *cer-xd.1455*, a sodium azide induced mutant in cultivar (cv.) Bonus, *cer-zv.268*, an ethylene imine induced mutant in cv. Foma and *nud1.a* a naturally occurring variant in cv. Himalaya, to cv. Bowman. For all three lines Bowman, the recurrent parent, was repeatedly backcrossed to obtain a cleaner background (Druka et al. 2011). Seeds of BW130, BW177, BW638 and Bowman were obtained from the James Hutton Institute. Seeds of *cer-xd.1455* in Bonus, *cer-zv.268* in Foma, Bonus and Foma were obtained from the NordGen genebank.

### Mapping

Plants of Bowman, BW130 and Bonus were genotyped using the barley 50 K iSelect *SNP* chip (Bayer et al. 2017). Markers were ordered based on barley cv. Morex genome assembly [Morex V3, (Mascher et al. 2017)] and plotted to visualize their position along the barley chromosomes using the ggplot2 R function (Wickham 2011). A BCF_2_ population was obtained from a BW130 X Bowman cross. 188 BCF2 lines were sown in 96-well plates and cereal compost under standard greenhouse conditions along with parents Bowman and BW130. Leaf samples were harvested and sent to LGC (Biosearch Technologies) for All-inclusive genotyping service (DNA extraction and *KASP* genotyping).

### Protein modeling, alignment and phylogenetic tree

The protein model of *HvGDSL1* was downloaded from Alphafold (Jumper et al. 2021, Varadi et al. 2022), after obtaining the UniProt ID number (F2DIQ7) by blasting *HvGDSL1* protein sequence at UniProt (UniProt Consortium 2023). A second model was predicted by Phyre2 (Kelley et al. 2015) based on homology with c3milA (isoamyl acetate-hydrolyzing esterase) from *Saccharomyces cerevisiae*.

To identify *HvGDSL1* orthologous sequences a BLASTP search was conducted using *HvGDSL1* protein sequence against the following databases: *Hordeum vulgare, Triticum aestivum, Brachypodium distachyon, Oryza sativa, Sorghum bicolor, Setaria italica, Zea mays, Agave tequilana, Solanum lycopersicum, Glycine max, Vitis vinifera, Theobroma cacao, Daucus carota, Beta vulgaris, Malus domestica, Arabidopsis thaliana, Liriodendron tulipifera, Thuja plicata, Selaginella moellendorffii, Physcomitrium patens, Marchantia polymorpha* and *Chlamydomonas reinhardtii*. The BLASTP search was conducted on Phytozome 13 and the genome versions used for each species are listed in **Supplementary Table S4**. Evolutionary analyses were conducted in MEGA X (Kumar et al. 2018). Full protein sequences were aligned using MUSCLE. The evolutionary history was inferred by using the Maximum Likelihood method and JTT matrix-based model (Jones et al. 1992). The tree with the highest log likelihood (−7,090.03) is shown. Initial tree(s) for the heuristic search were obtained automatically by applying Neighbor-Joining and BioNJ algorithms to a matrix of pairwise distances estimated using the JTT model, and then selecting the topology with superior log likelihood value. The tree is drawn to scale with branch lengths measured in the number of substitutions per site. This analysis involved 24 amino acid sequences. There were a total of 376 positions in the final dataset. A Clustal omega alignment of the *GDSL* protein containing the conserved terminal domain was conducted in the EMBL-EBI website (Madeira et al. 2022).

### Cuticle permeability assessment

Plants were grown in 24-well trays in soil under greenhouse conditions: 16-hour light photoperiod, temperature day/night 18/14⁰C. Additional light and heating were applied when needed.

For the TB test, 4-cm leaf segments from the central part of the second leaf of 3-week-old plants were immersed in 0.05% TB aqueous solution for 2 h. Subsequently, leaf segments were rinsed with water to remove the excess TB, before being pat-dried and photographed. The experiment was repeated on four plants per genotype.

For the water loss test, 2-cm leaf segments from the central part of the second leaf of 2-week-old plants were placed on a petri dish. Petri dishes were weighed to record fresh weight. Subsequently, open petri dishes were placed in an oven at 30⁰C and weight was recorded every hour for 7 h. After that the leaf segments were left for 24 h at 50⁰C to fully dry them and the dried weight was recorded. For each genotype, four biological replicates were measured. Water loss at each timepoint is calculated as percentage of the total leaf water content and plotted using the ggplot2 R function (Wickham 2011). Area under the curve was calculated for each genotype, and ANOVA was used to check for statistical significance among genotypes.

### Skinning index and hull separation

For the visual identification of the skinning phenotype, plants of Bowman, BW130 and BW638 were grown under a polytunnel during spring/summer 2016 in Dundee, Scotland. At maturity, the seed from single plants was harvested and threshed with a Wintersteiger pedal thresher. A variable number between 20 and 100 seeds was visually screened for seed defects from three plants for each genotype. Any seed presenting at least 20% of hull removed was considered ‘skinned’. Pictures of representative seeds from each genotype were taken with a Canon EOS 750D. To quantify hull attachment, plants of Bowman and BW130 were grown in a polytunnel for two seasons (spring/summer 2018 and spring summer 2019) in Dundee, Scotland. Single plants were harvested at maturity and threshed with a Haldrup single ear thresher. Per each sample, 25 g of seed were run through a Lab Scale Debranner TM05 (Satake) for 15 s and the separated hull was weighed. Subsequently samples were run through the Debranner for an additional 15 s (pearled grain) and the separated hull was weighed. This latter summed to the hull obtained after 15 s is considered the total hull. Ratio was calculated between the hull separated after 15 s and the total hull, and ANOVA was used to check for statistical significance between genotypes.

### SB staining

Plants were grown in 24-well trays in soil under greenhouse conditions: 16-h light photoperiod, temperature day/night 18/14⁰C. Additional light and heating were applied when needed. At heading one central spikelet from the main stem was dissected to expose floral organs. Anthesis was defined when the anthers were yellow and dehiscing pollen and the carpel was just beginning to elongate, the spike was marked. In the following days spikes were collected at regular intervals from 9 to 18  DPA, the hull was carefully removed, and exposed caryopses were stored at −20⁰C. Samples were thawed and immersed in 1 mL of 0.1% (w/v) SB in 70% ethanol for 10 min. Subsequently, samples were immersed in 50% (v/v) ethanol for 2 min to remove the excess non-bound dye, before being pat-dried and photographed. The experiment was repeated on three plants per genotype per each timepoint. Image J was used to quantify the total area and the SB stained area on the dorsal side of the caryopsis. ANOVA was used to check for statistical significance among genotypes at each timepoint.

### Microscopy

Plants were grown and screened for anthesis as described for the SB staining experiment. For SEM, caryopsis segments (4 mm long × 4 mm wide × 1 mm thick) were cut from the center of the dorsal side of dehulled caryopses of 7 and 11 DPA. Samples were prepared according to Brennan et al. (2017a) with some modifications. For fixation, segments were fixed in 4% (w/v) paraformaldehyde and 2% (w/v) glutaraldehyde in 100 mM sodium 1,4-piperazinediethanesulfonic acid (PIPES) buffer (pH 7.2) for 4 h at room temperature, then left overnight at 4°C. Fixed tissue was washed three times in 0.1 m sodium cacodylate buffer (pH 7.3) for 10 min each time and then was stored at 4°C. Tissue was then post-fixed in 1% OsO4 with 1.5% Na ferrocyanide in cacodylate buffer for 60 min, followed by two washes in 0.5 mL 0.1 m sodium cacodylate (pH 5) for 10 min each time. Post-fixed samples were dehydrated in an alcohol series of 50%, 70%, 80%, 90%, 95% and 100% repeated for 10 min each and were transferred into 100% ethanol with molecular sieves to wash twice for 10 min each. Dehydrated samples were transferred into a critical point dryer filled with 100% ethanol (molecular sieves treated) and critical point dried. Dried specimens were mounted on aluminum stubs, placed in a sputter coater for Au/Pd (10 nm) coating before being imaged on a JEOL JSM 7400 F SEM.

For TEM, a central segment (3 mm long × 1 mm wide × 1 mm thick) was sliced out of Bowman, BW130, BW177 and BW638 caryopses at 11 DPA. These segments included all maternal cell layers and endosperm but avoided the area over the embryo which does not produce a cementing layer. Samples were subjected to fixation and post-fixation as described for SEM samples. Post-fixed samples were subsequently stained with 1% uranyl acetate in acetate buffer for 1 h and then dehydrated in alcohol series from 50% to 100% for 10 min in each followed by in 100% propylene oxide twice for 10 min each time. A 1:1 mix of 50% propylene oxide and 50% Durcupan resin was added to samples, then rotated overnight before removing the cap for propylene oxide to evaporate for 2–3 h. Samples were then put in 100% Durcupan resin in specimen bottle caps for 5 h and then were changed to fresh Durcupan resin in capsules and polymerized at 60°C overnight. Sections of 70–100 nm thickness were cut on Leica Ultracut UCT and were stained with 3% uranyl acetate followed by Reynolds lead citrate (1.33 g lead nitrate + 1.76 g sodium citrate + 8 mL 1 N NaOH in 50 mL water) for 10 min each. Finally, these sections were imaged on JEOL 1200EX TEM using SIS Megaview III camera. Image J was used to measure the thickness of the cuticle. For each biological replicate, 11 images were taken (except Bowman replicate 1, with 7 images) and each image was measured in three points and averaged. Kruskal–Wallis ANOVA was used to check for statistical significance among genotypes within each replicate.

For leaf impressions, 2 cm fragments of the second leaf of two weeks old plants were collected and placed on double sided tape, abaxial side up. Leaf fragments were covered with transparent nail polish and let dry for few minutes. Cello tape was used to transfer the nail polish impressions from the leaf surface to a microscopy slide. Slides were imaged with a Zeiss Axioscop2 Plus and images were collected with a Zeiss Axiocam 506C at 100× or 200× magnifications. Image J functions embedded in Omero were used to take measurements. For stomata density, three cell files per genotype were counted; for stomata size, 24 stomata per genotype were measured. Significant differences were assigned following a paired *t*-test.

### Wax and cutin extraction from grain

Plants were grown and screened for anthesis as described for the SB staining experiment. Spikes were collected at 5 and 11 DPA, the hull was carefully removed, and hull and exposed caryopses were separately stored at −80⁰C. Surface waxes were extracted from 9–15 caryopses and their hull for each replicate (3 replicates per genotype) by dipping the sample for 1 min in 10 mL dichloromethane (DCM) containing 10 µg methyl-nonadecanoate as the internal standard and dried with a combination of heat and nitrogen. Extracts were derivatized by resuspending them in 80 µL *N*-*O*-bis-trimethylsilyltrifluoroacetamide (BSTFA) and incubating at 140°C for 1 h. Wax components were identified using Gas Chromatography–Mass Spectrometry (GC–MS) using a Trace DSQTM II Series Quadrupole system (Thermo Electron Corporation, Hemel Hempstead, UK), fitted with a CTC CombiPAL autosampler (CTC Analytics, Switzerland). Samples were analyzed as previously described (Brennan et al. 2017a) with the following modifications: the programmable temperature vaporizing (PTV) injector operated in split mode (40:1 ratio), and solvent delay for mass spectrum acquisition was 2.8 min. Data were acquired and analyzed using Xcalibur™ (version 2.0.7, Thermo Fisher Scientific): specific ions, characteristic of each compound, were selected and used for compound detection and quantification in a processing method. Processed data were manually checked and corrected where necessary. Values were considered only if they were at least 25% higher than their average value in the blank samples and the average blank value was subtracted. Compounds found in only one replicate were assigned as ‘traces’.

The same samples used for wax extraction were exhaustively extracted with DCM over a period of 2–3 weeks with frequent change of solvent, subsequently dried and kept under vacuum. Cutin was extracted following (Franke et al. 2005) with some modifications. Briefly, cutin monomers were solubilized with 2 mL of 1 N MeOH/HCL for 2 h at 80℃, shaking occasionally. A 20 µg of dotriacontane were added to each sample as internal standard. After terminating the reaction with 2 mL of saturated NaCl, samples were extracted 3 times with 2 mL hexane. The three extracts were combined, evaporated in a vacuum evaporator and derivatized with 20 µL BSTFA, 20 µL of pyridine and 50 µL of chloroform for 60 min at 70℃. Cutin components were identified on representative samples with GC–MS using an Agilent 7890 A gas chromatograph combined with an Agilent (Agilent Technologies, Germany) 5975C quadrupole mass selective detector. Separation was achieved using a HP-5 MS, 30 metre × 0.32 mm, 0.25 µm column (Agilent Technologies). Extracted sample solutions (1µL) were injected with a 20:1 split-injection at 280℃, oven 2 min at 50℃, 10℃ min-1–150℃, 1 min at 150℃, 3℃ min-1 to 310℃, 15 min at 310℃ and He carrier gas at a constant flow rate of 3.0 mL min-1. Cutin components were identified based on their Electron Ionization Mass Spectra. Quantitative determination of cutin components was carried out with an identical GC-system equipped with a flame ionization detector based on the internal standard, except split-injection was 5:1 at 320 ℃. For both the wax and cutin components ANOVA was used to check for statistical significance among genotypes within each developmental stage or between developmental stages within a genotype.

### Gene quantitative expression

Plants were grown and screened for anthesis as described for the SB staining experiment. Ten dehulled caryopses of 3, 5, 7, 9 and 11 DPA from each genotype were pooled as one bioreplicate, flash-frozen in liquid nitrogen and stored at −80°C; three bio-replicates per genotype per stage were harvested. RNA isolation was carried out using TRI reagents (Sigma-Aldrich, Darmstadt, Germany) following manufacturer’s recommendations with an additional chloroform extraction. RNA concentration and quality was assessed on a Nanodrop 2000 (Thermo Scientific). cDNA synthesis was performed using the ProtoScript® II First Strand cDNA Synthesis Kit and 1 μg total RNA.

The FastStart Universal Probe Master mix (Roche, Basel, Switzerland) coupled with the Universal Probe Library probes or the SYBR Green Power Up kit (Thermo Fisher Scientific, Waltham, MA, USA) were used to detect transcripts of target and reference genes. The primers used for qRT-PCR are listed in **Supplementary Table S13**. Three biological and three technical replicates were used for the quantification of each target. The reactions were run on the Applied Biosystems StepOne system. The expression values were determined using the 2^–ΔΔCT^ method relative to one Bowman replicate to present the expression levels across the genotypes and stages.

### In situ hybridization

Plants were grown and screened for anthesis as described for the SB staining experiment. Caryopses were harvested at 5- and 9-DPA and fixed in 4% formaldehyde in PEM Buffer [0.1 m PIPES (pH 6.95), 1 mM EGTA, 1 mM MgSO4]. Tissues were processed in a Leica TP1020 automated tissue processor with the following steps: 1 h 70% ethanol, 1 h 30 min in 80% ethanol, 90% ethanol for 2 h, 100% ethanol for 1 h, 100% ethanol for 1 h 30 min, 100% ethanol for 2 h, 100% xylene for 1 h, 100% xylene for 1 h 30 min, Paraplast® wax at 65°C for 2 h and a final step of Paraplast® wax at 65°C for 4 h. Samples were embedded using a Leica EG1160 wax embedder and sectioned using a Leica RM2265 microtome at 8 µm thickness. Probe synthesis, slide processing and in situ hybridization were done as described in Gramma and Wahl (2023). *HvGDSL1* sense and antisense probe templates were generated as described in Li et al. (2017) with both probes having the T7 promoter. Primers for probe template generation are in **Supplementary Table S13**. Slides were imaged using a Zeiss axioscan 7 slide scanner at 20× magnification by the Dundee Imaging Facility.

### Haplotype analysis

*SNP* data of HORVU.MOREX.r3.4 HG0357220 were retrieved from published exome-capture datasets of *H. spontaneum* and *H. vulgare* lines (Russell et al. 2016, Bustos‐Korts et al. 2019). The dataset was filtered to retain sites with ≥98% of samples homozygous. Accessions with missing data points or heterozygosity at these sites were excluded. The resulting dataset, including 477 accessions, was used to build a haplotype network. Median-Joining haplotype network construction was performed using *PopArt* [*popart.otago.ac.nz* (Leigh et al. 2015)]. Haplotypes were plotted on a world map using the rworldmap R package [v1.3–6; (South 2011)]. Where available, latitude and longitude of sampling or based on subnational production mid-point as in Russell et al. (2016) were used. If unknown, the latitude and longitude of the capital city of the country of origin were used. This information was not available for 13 lines which were therefore excluded from this analysis.

## Supplementary Material

pcae041_Supp

## Data Availability

Large datasets were not generated; however, all scripts and raw data are available upon request.

## References

[R1] Aharoni A., Dixit S., Jetter R., Thoenes E., van Arkel G. and Pereira A. (2004) The SHINE clade of AP2 domain transcription factors activates wax biosynthesis, alters cuticle properties, and confers drought tolerance when overexpressed in Arabidopsis. *Plant Cell* 16: 2463–2480. 15319479 10.1105/tpc.104.022897 PMC520946

[R2] Akoh C.C., Lee G.-C., Liaw Y.-C., Huang T.-H. and Shaw J.-F. (2004) GDSL family of serine esterases/lipases. *Prog. Lipid Res*. 43: 534–552. 15522763 10.1016/j.plipres.2004.09.002

[R3] Anderson A.C., Stangherlin S., Pimentel K.N., Weadge J.T. and Clarke A.J. (2022) The SGNH hydrolase family: a template for carbohydrate diversity. *Glycobiology* 32: 826–848. 35871440 10.1093/glycob/cwac045 PMC9487903

[R4] Bayer M.M., Rapazote-Flores P., Ganal M., Hedley P.E., Macaulay M., Plieske J., et al. (2017) Development and evaluation of a barley 50k iSelect SNP array. *Front. Plant Sci*. 8: 1792. 10.3389/fpls.2017.01792 PMC5651081 29089957

[R5] Bird D., Beisson F., Brigham A., Shin J., Greer S., Jetter R., et al. (2007) Characterization of Arabidopsis ABCG11/WBC11, an ATP binding cassette (ABC) transporter that is required for cuticular lipid secretion. *Plant J*. 52: 485–498. 17727615 10.1111/j.1365-313X.2007.03252.x

[R6] Bourdenx B., Bernard A., Domergue F., Pascal S., Léger A., Roby D., et al. (2011) Overexpression of Arabidopsis ECERIFERUM1 promotes wax very-long-chain alkane biosynthesis and influences plant response to biotic and abiotic stresses. *Plant Physiol*. 156: 29–45. 21386033 10.1104/pp.111.172320 PMC3091054

[R7] Brennan M., Hedley P.E., Topp C.F., Morris J., Ramsay L., Mitchell S., et al. (2019) Development and quality of barley husk adhesion correlates with changes in caryopsis cuticle biosynthesis and composition. *Front. Plant Sci*. 10: 672. 10.3389/fpls.2019.00672 PMC6543523 31178883

[R8] Brennan M., Shepherd T., Mitchell S., Topp C. and Hoad S. (2017a) Husk to caryopsis adhesion in barley is influenced by pre-and post-anthesis temperatures through changes in a cuticular cementing layer on the caryopsis. *BMC Plant Biol*. 17: 1–19. 29058624 10.1186/s12870-017-1113-4 PMC5651604

[R9] Brennan M., Topp C.F.E. and Hoad S.P. (2017b) Variation in grain skinning among spring barley varieties induced by a controlled environment misting screen. *J. Agric. Sci*. 155: 317–325.

[R10] Brick D.J., Brumlik M.J., Buckley J.T., Cao J.-X., Davies P.C., Misra S., et al. (1995) A new family of lipolytic plant enzymes with members in rice, arabidopsis and maize. *FEBS Lett*. 377: 475–480. 8549779 10.1016/0014-5793(95)01405-5

[R11] Bryce J.H., Goodfellow V., Agu R.C., Brosnan J.M., Bringhurst T.A. and Jack F.R. (2010) Effect of different steeping conditions on endosperm modification and quality of distilling malt. *J. Inst. Brew*. 116: 125–133.

[R12] Bustos‐Korts D., Dawson I.K., Russell J., Tondelli A., Guerra D., Ferrandi C., et al. (2019) Exome sequences and multi‐environment field trials elucidate the genetic basis of adaptation in barley. *Plant J*. 99: 1172–1191. 31108005 10.1111/tpj.14414 PMC6851764

[R13] Chen G., Komatsuda T., Ma J.F., Nawrath C., Pourkheirandish M., Tagiri A., et al. (2011) An ATP-binding cassette subfamily G full transporter is essential for the retention of leaf water in both wild barley and rice. *Proc. Natl. Acad. Sci*. 108: 12354–12359. 21737747 10.1073/pnas.1108444108 PMC3145689

[R14] Ding L.-N., Li M., Wang W.-J., Cao J., Wang Z., Zhu K.-M., et al. (2019) Advances in plant GDSL lipases: from sequences to functional mechanisms. *Acta Physiol. Plant* 41: 1–11.

[R15] Druka A., Franckowiak J., Lundqvist U., Bonar N., Alexander J., Houston K., et al. (2011) Genetic dissection of barley morphology and development. *Plant PhysiologyPlant Physiol*. 155: 617–627. 10.1104/pp.110.166249 PMC3032454 21088227

[R16] Franke R., Briesen I., Wojciechowski T., Faust A., Yephremov A., Nawrath C., et al. (2005) Apoplastic polyesters in Arabidopsis surface tissues–a typical suberin and a particular cutin. *Phytochemistry* 66: 2643–2658. 16289150 10.1016/j.phytochem.2005.09.027

[R17] Gaines R.L., Bechtel D.B. and Pomeranz Y. (1985) A microscopic study on the development of a layer in barley that causes hull-caryopsis adherence. *Cereal Chem*. 62: 35–40.

[R18] Girard A.-L., Mounet F., Lemaire-Chamley M., Gaillard C., Elmorjani K., Vivancos J., et al. (2012) Tomato GDSL1 is required for cutin deposition in the fruit cuticle. *Plant Cell* 24: 3119–3134. 22805434 10.1105/tpc.112.101055 PMC3426136

[R19] Gramma V. Wahl V. (2023) RNA in situ hybridization on plant tissue sections: expression analysis at cellular resolution. *In* *Flower Development: Methods and Protocols*. Edited by Riechmann, J.L. and Ferrándiz, C. pp. 331–350 . Humana: New York, NY, USA. 10.1007/978-1-0716-3299-4_17 37540368

[R20] Harlan H.V. (1920) Daily development of kernels of hannchen barley from flowering to maturity, at Aberdeen, Idaho. *J. Agric. Res*. 19: 393–429.

[R21] Harlan J. (1968) Barley: Origin, Botany, Culture, Winterhardiness, Genetics, Utilization, Pests. Agriculture Handbook No. 338. US Department of Agriculture, Washington, DC.

[R22] Hen-Avivi S., Savin O., Racovita R.C., Lee W.-S., Adamski N.M., Malitsky S., et al. (2016) A metabolic gene cluster in the wheat W1 and the barley Cer-cqu loci determines β-diketone biosynthesis and glaucousness. *Plant Cell* 28: 1440–1460. 27225753 10.1105/tpc.16.00197 PMC4944414

[R23] Hong L., Brown J., Segerson N.A., Rose J.K. and Roeder A.H. (2017) CUTIN SYNTHASE 2 maintains progressively developing cuticular ridges in Arabidopsis sepals. *Mol. Plant* 10: 560–574. 28110092 10.1016/j.molp.2017.01.002

[R24] Jenks M.A., Rashotte A.M., Tuttle H.A. and Feldmann K.A. (1996) Mutants in Arabidopsis thaliana altered in epicuticular wax and leaf morphology. *Plant Physiol*. 110: 377–385. 12226189 10.1104/pp.110.2.377 PMC157730

[R25] Jones D.T., Taylor W.R. and Thornton J.M. (1992) The rapid generation of mutation data matrices from protein sequences. *Bioinformatics* 8: 275–282. 10.1093/bioinformatics/8.3.275 1633570

[R26] Jumper J., Evans R., Pritzel A., Green T., Figurnov M., Ronneberger O., et al. (2021) Highly accurate protein structure prediction with AlphaFold. *Nature* 596: 583–589. 34265844 10.1038/s41586-021-03819-2 PMC8371605

[R27] Kannangara R., Branigan C., Liu Y., Penfield T., Rao V., Mouille G., et al. (2007) The transcription factor WIN1/SHN1 regulates cutin biosynthesis in Arabidopsis thaliana. *Plant Cell* 19: 1278–1294. 17449808 10.1105/tpc.106.047076 PMC1913754

[R28] Kelley L.A., Mezulis S., Yates C.M., Wass M.N. and Sternberg M.J. (2015) The Phyre2 web portal for protein modeling, prediction and analysis. *Nat. Protoc*. 10: 845–858. 25950237 10.1038/nprot.2015.053 PMC5298202

[R29] Kumar S., Stecher G., Li M., Knyaz C., Tamura K. and Battistuzzi F.U. (2018) MEGA X: molecular evolutionary genetics analysis across computing platforms. *Mol. Biol. Evol*. 35: 1547–1549. 29722887 10.1093/molbev/msy096 PMC5967553

[R30] Leigh J.W., Bryant D. and Nakagawa S. (2015) popart: full-feature software for haplotype network construction. *Meth. Ecol. Evol*. 6: 1110–1116.

[R31] Li C., Chen G., Mishina K., Yamaji N., Ma J.F., Yukuhiro F., et al. (2017) A GDSL‐motif esterase/acyltransferase/lipase is responsible for leaf water retention in barley. *Plant Direct* 1: e00025. 10.1002/pld3.25 PMC6508521 31245672

[R32] Li C., Haslam T.M., Krüger A., Schneider L.M., Mishina K., Samuels L., et al. (2018) The β-ketoacyl-CoA synthase Hv KCS1, encoded by Cer-zh, plays a key role in synthesis of barley leaf wax and germination of barley powdery mildew. *Plant Cell Physiol*. 59: 811–827. 10.1093/pcp/pcy020 29401261

[R33] Li C., Liu C., Ma X., Wang A., Duan R., Nawrath C., et al. (2015) Characterization and genetic mapping of eceriferum-ym (cer-ym), a cutin deficient barley mutant with impaired leaf water retention capacity. *Breed. Sci*. 65: 327–332. 26366115 10.1270/jsbbs.65.327 PMC4542933

[R35] Li C., Wang A., Ma X., Pourkheirandish M., Sakuma S., Wang N., et al. (2013) An eceriferum locus, cer-zv, is associated with a defect in cutin responsible for water retention in barley (Hordeum vulgare) leaves. *Theor. Appl. Genet*. 126: 637–646. 23124432 10.1007/s00122-012-2007-3

[R34] Liu X., Bourgault R., Galli M., Strable J., Chen Z., Feng F., et al. (2021) The FUSED LEAVES1‐ADHERENT1 regulatory module is required for maize cuticle development and organ separation. *New Phytol*. 229: 388–402. 32738820 10.1111/nph.16837 PMC7754373

[R36] Lundqvist U. and Franckowiak J. (1996) Barley genetic newsletter.

[R37] Lundqvist U. and Lundqvist A. (1988) Mutagen specificity in barley for 1580 eceriferum mutants localized to 79 loci. *Hereditas* 108: 1–12.

[R38] Madeira F., Pearce M., Tivey A.R., Basutkar P., Lee J., Edbali O., et al. (2022) Search and sequence analysis tools services from EMBL-EBI in 2022. *Nucleic Acids Res*. 50: W276–W279. 35412617 10.1093/nar/gkac240 PMC9252731

[R39] Mascher M., Gundlach H., Himmelbach A., Beier S., Twardziok S.O. and Wicker T. (2017) A chromosome conformation capture ordered sequence of the barley genome. *Nature* 544: 427–433. 28447635 10.1038/nature22043

[R40] Milne L., Bayer M., Rapazote-Flores P., Mayer C.-D., Waugh R. and Simpson C.G. (2021) EORNA, a barley gene and transcript abundance database. *Sci. Data* 8: 90. 10.1038/s41597-021-00872-4 PMC7994555 33767193

[R41] Nadakuduti S.S., Pollard M., Kosma D.K., Allen C. Jr, Ohlrogge J.B. and Barry C.S. (2012) Pleiotropic phenotypes of the sticky peel mutant provide new insight into the role of CUTIN DEFICIENT2 in epidermal cell function in tomato. *Plant Physiol*. 159: 945–960. 22623518 10.1104/pp.112.198374 PMC3387719

[R42] Newman C. and Newman R.K. (2006) A brief history of barley foods. *Cereal Foods World* 51: 4–7.

[R43] Nice L.M., Steffenson B.J., Blake T.K., Horsley R.D., Smith K.P. and Muehlbauer G.J. (2017) Mapping agronomic traits in a wild barley advanced backcross–nested association mapping population. *Crop Sci*. 57: 1199–1210.

[R44] Panikashvili D., Shi J.X., Schreiber L. and Aharoni A. (2011) The Arabidopsis ABCG13 transporter is required for flower cuticle secretion and patterning of the petal epidermis. *New Phytol*. 190: 113–124. 21232060 10.1111/j.1469-8137.2010.03608.x

[R45] Park -J.-J., Jin P., Yoon J., Yang J.-I., Jeong H.J., Ranathunge K., et al. (2010) Mutation in Wilted Dwarf and Lethal 1 (WDL1) causes abnormal cuticle formation and rapid water loss in rice. *Plant Mol. Biol*. 74: 91–103. 20593223 10.1007/s11103-010-9656-x

[R46] Radchuk V., Weier D., Radchuk R., Weschke W. and Weber H. (2011) Development of maternal seed tissue in barley is mediated by regulated cell expansion and cell disintegration and coordinated with endosperm growth. *J. Exp. Bot*. 62: 1217–1227. 21059741 10.1093/jxb/erq348 PMC3022404

[R47] Richardson A., Wojciechowski T., Franke R., Schreiber L., Kerstiens G., Jarvis M., et al. (2007) Cuticular permeance in relation to wax and cutin development along the growing barley (Hordeum vulgare) leaf. *Planta* 225: 1471–1481. 17171372 10.1007/s00425-006-0456-0

[R48] Rowland O., Lee R., Franke R., Schreiber L. and Kunst L. (2007) The CER3 wax biosynthetic gene from Arabidopsis thaliana is allelic to WAX2/YRE/FLP1. *FEBS Lett*. 581: 3538–3544. 17624331 10.1016/j.febslet.2007.06.065

[R49] Russell J., Mascher M., Dawson I.K., Kyriakidis S., Calixto C., Freund F., et al. (2016) Exome sequencing of geographically diverse barley landraces and wild relatives gives insights into environmental adaptation. *Nat. Genet*. 48: 1024–1030. 27428750 10.1038/ng.3612

[R50] Samuels L., Kunst L. and Jetter R. (2008) Sealing plant surfaces: cuticular wax formation by epidermal cells. *Annu. Rev. Plant Biol*. 59: 683–707. 18251711 10.1146/annurev.arplant.59.103006.093219

[R51] Schneider L.M., Adamski N.M., Christensen C.E., Stuart D.B., Vautrin S., Hansson M., et al. (2016) The Cer-cqu gene cluster determines three key players in a β-diketone synthase polyketide pathway synthesizing aliphatics in epicuticular waxes. *J. Exp. Bot*. 67: 2715–2730. 26962211 10.1093/jxb/erw105 PMC4861019

[R52] Segado P., Heredia-Guerrero J.A., Heredia A. and Domínguez E. (2020) Cutinsomes and CUTIN SYNTHASE1 function sequentially in tomato fruit cutin deposition. *Plant Physiol*. 183: 1622–1637. 32457092 10.1104/pp.20.00516 PMC7401130

[R53] Shen G., Sun W., Chen Z., Shi L., Hong J. and Shi J. (2022) Plant GDSL esterases/lipases: evolutionary, physiological and molecular functions in plant development. *Plants* 11: 468. 10.3390/plants11040468 PMC8880598 35214802

[R54] South A. (2011) rworldmap: a new R package for mapping global data. *R J*. 3: 35–43.

[R55] Su R., Chen L., Wang Z. and Hu Y. (2020) Differential response of cuticular wax and photosynthetic capacity by glaucous and non-glaucous wheat cultivars under mild and severe droughts. *Plant Physiol. Biochem*. 147: 303–312. 31901453 10.1016/j.plaphy.2019.12.036

[R56] Taketa S., Amano S., Tsujino Y., Sato T., Saisho D., Kakeda K., et al. (2008) Barley grain with adhering hulls is controlled by an ERF family transcription factor gene regulating a lipid biosynthesis pathway. *Proc. Natl. Acad. Sci. U.S.A*. 105: 4062–4067. 18316719 10.1073/pnas.0711034105 PMC2268812

[R57] UniProt Consortium . (2023) UniProt: the universal protein knowledgebase in 2023. *Nucleic Acids Res*. 51: D523–D531. 36408920 10.1093/nar/gkac1052 PMC9825514

[R58] Varadi M., Anyango S., Deshpande M., Nair S., Natassia C., Yordanova G., et al. (2022) AlphaFold protein structure database: massively expanding the structural coverage of protein-sequence space with high-accuracy models. *Nucleic Acids Res*. 50: D439–D444. 34791371 10.1093/nar/gkab1061 PMC8728224

[R59] von Wettstein-knowles P. (2017) The polyketide components of waxes and the Cer-cqu gene cluster encoding a novel polyketide synthase, the β-diketone synthase, DKS. *Plants* 6: 28. 10.3390/plants6030028 PMC5620584 28698520

[R60] Weidenbach D., Jansen M., Franke R.B., Hensel G., Weissgerber W., Ulferts S., et al. (2014) Evolutionary conserved function of barley and Arabidopsis 3-KETOACYL-CoA SYNTHASES in providing wax signals for germination of powdery mildew fungi. *Plant Physiol*. 166: 1621–1633. 25201879 10.1104/pp.114.246348 PMC4226380

[R61] Wickham H. (2011) Wiley interdisciplinary reviews: computational statistics. *Ggplo 2* 3: 180–185.

[R62] Yeats T.H. and Rose J.K. (2013) The formation and function of plant cuticles. *Plant Physiol*. 163: 5–20. 23893170 10.1104/pp.113.222737 PMC3762664

[R63] Zhang Z., Wang W., Li W. and Zhang T. (2013) Genetic interactions underlying the biosynthesis and inhibition of β-diketones in wheat and their impact on glaucousness and cuticle permeability. *PLoS one* 8: e54129. 10.1371/journal.pone.0054129 PMC3547958 23349804

