# Supplementary material for: A GDSL-motif Esterase/Lipase Affects Wax and Cutin Deposition and Controls Hull-Caryopsis Attachment in Barley
Source: Plant Cell Physiol. 2024 Apr 26;65(6):999–1013. doi: 10.1093/pcp/pcae041 (PMC11209556; doi:10.1093/pcp/pcae041)
Supplement: pcae041_Supp [file pcae041_supp.zip › suppl_data/pcp-2023-e-00309-File009.pdf]

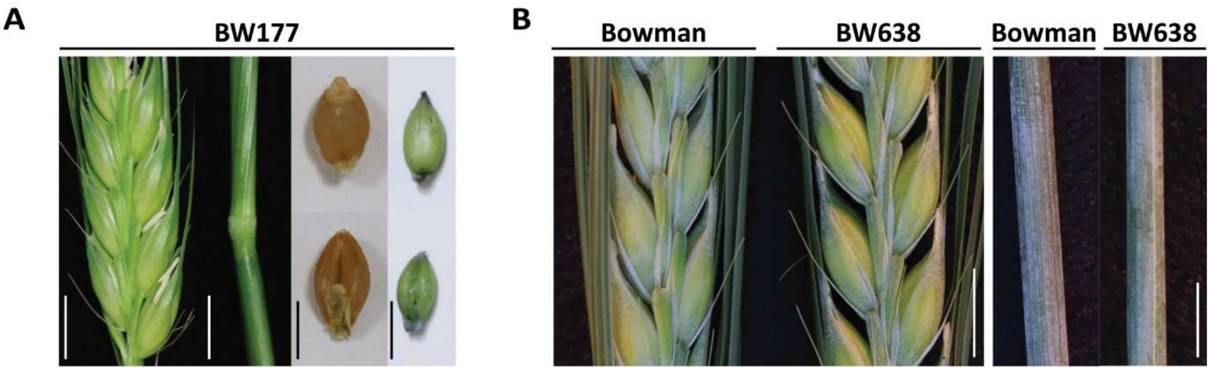

Supplementary figure S1. Phenotypes of BW177 and BW638. **A**, BW177 carries the *cer-zv.268* allele and shows spike and stem lacking the wax bloom, naked grain, and 18 days post anthesis caryopses that do not stain with Sudan Black B. Scale bars = 5mm. **B**, Wax bloom deposition on spikes and leaf sheaths of Bowman and BW638. Scale bars = 5 mm.

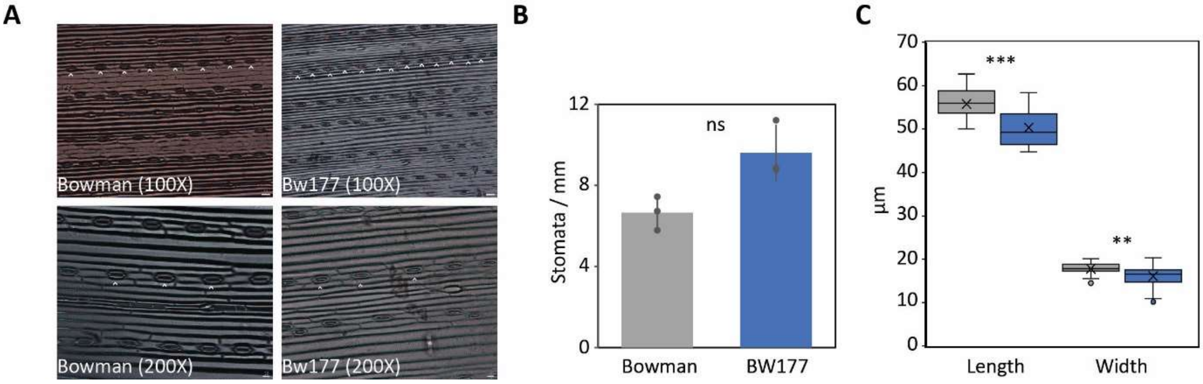

Supplementary Figure S2. Stomata density and size of Bowman and BW177. **A**, Abaxial leaf impressions from the second leaf of Bowman and BW177. Scale bars = 50µm (100X magnification); 20 µm (200X magnification). Arrows indicate stomata. **B**, Average number of stomata per mm along a stomata file on the abaxial side shown for Bowman and BW177. Dots represent values from individual cell files. Error bars represent standard deviation. ns = not significant (paired T-test). **C**, Length and width of stomata of Bowman (grey) and BW177 (blue). *** = p-value < 0.001; ** = p-value < 0.01 (paired T-test); N=24.

A

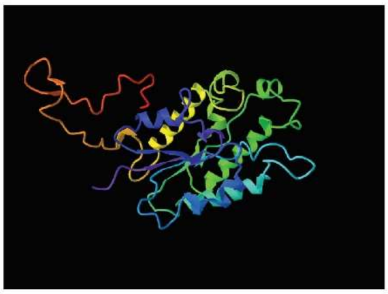

B

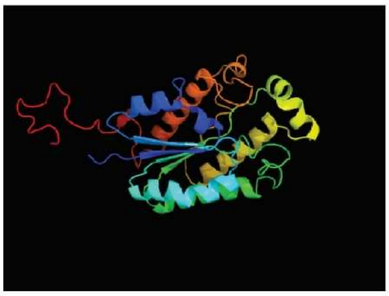

C

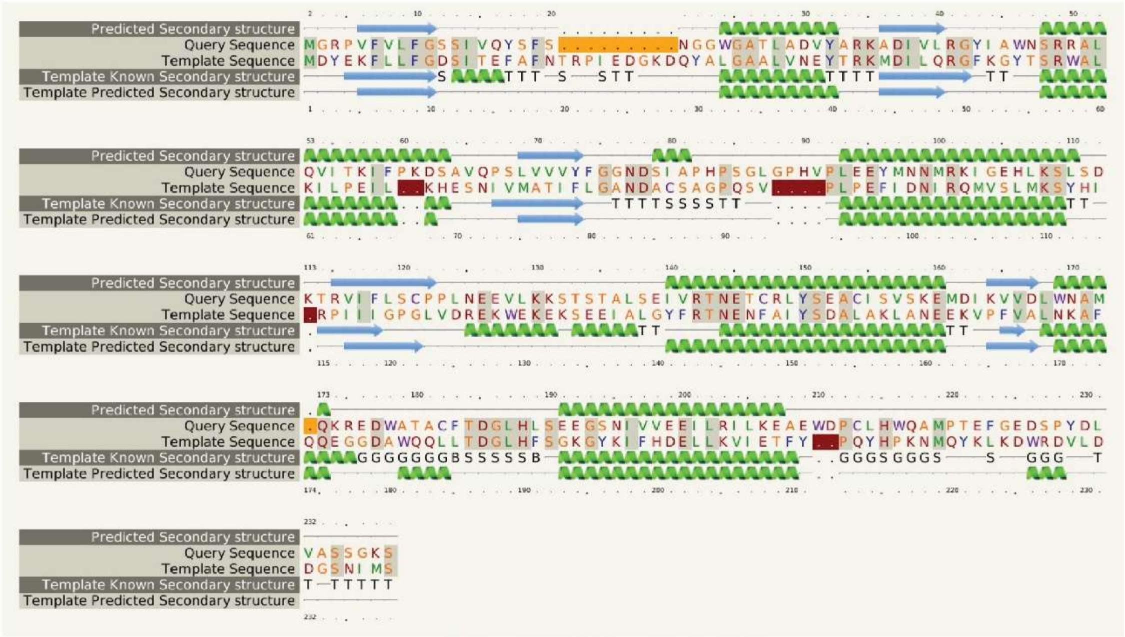
Predicted Secondary structure
Query Sequence
Template Sequence
Template Known Secondary structure
Template Predicted Secondary structure

D

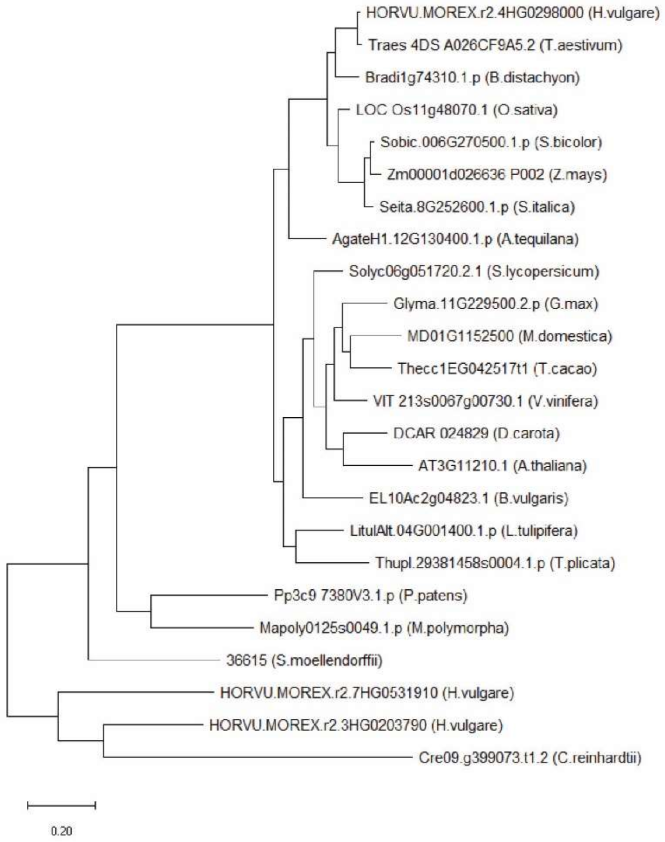
HORVU.MOREX.r2.4HG0298000 (H.vulgare)
Traes 4DS A026CF9A5.2 (T.aestivum)
Bradi1g74310.1.p (B.distachyon)
LOC Os11g48070.1 (O.sativa)
Sobic.006G270500.1.p (S.bicolor)
Zm00001d026636 P002 (Z.mays)
Seita.8G252600.1.p (S.italica)
AgateH1.12G130400.1.p (A.tequilana)
Solyc06g051720.2.1 (S.lycopersicum)
Glyma.11G229500.2.p (G.max)
MD01G1152500 (M.domestica)
Thecc1EG042517t1 (T.cacao)
VIT 213s0067g00730.1 (V.vinifera)
DCAR 024829 (D.carota)
AT3G11210.1 (A.thaliana)
EL10Ac2g04823.1 (B.vulgaris)
LitulAlt.04G001400.1.p (L.tulipifera)
Thupl.29381458s0004.1.p (T.plicata)
Pp3c9 7380V3.1.p (P.patens)
Mapoly0125s0049.1.p (M.polymorpha)
36615 (S.moellendorffii)
HORVU.MOREX.r2.7HG0531910 (H.vulgare)
HORVU.MOREX.r2.3HG0203790 (H.vulgare)
Cre09.g399073.t1.2 (C.reinhardtii)
0.20

E

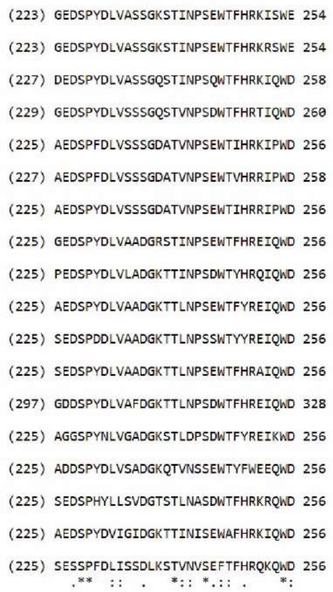
(223) GEDSPYDLVASSGKSTINPSEWTFHRKISWE 254
(223) GEDSPYDLVASSGKSTINPSEWTFHRKRSWE 254
(227) DEDSPYDLVASSGQSTINPSQWTFHRKIQWD 258
(229) GEDSPYDLVSSSGQSTVNPSDWTFHRTIQWD 260
(225) AEDSPFDLVSSSGDATVNPSEWTIHRKIPWD 256
(227) AEDSPFDLVSSSGDATVNPSEWTVHRRIPWD 258
(225) AEDSPYDLVSSSGDATVNPSEWTIHRRIPWD 256
(225) GEDSPYDLVAADGRSTINPSEWTFHREIQWD 256
(225) PEDSPYDLVLADGKTTINPSDWTYHRQIQWD 256
(225) AEDSPYDLVAADGKTTLNPSEWTFYREIQWD 256
(225) SEDSPDDLVAADGKTTLNPSSWTYYREIQWD 256
(225) SEDSPYDLVAADGKTTLNPSEWTFHRAIQWD 256
(297) GDDSPYDLVAFDGKTTLNPSDWTFHREIQWD 328
(225) AGGSPYNLVGADGKSTLDPSDWTFYREIKWD 256
(225) ADDSPYDLVSADGKQTVNSSEWTYFWEEQWD 256
(225) SEDSPHYLLSVDGTSTLNASDWTFHRKRQWD 256
(225) AEDSPYDVIGIDGKTTINISEWAFHRKIQWD 256
(225) SESSPFDLISSDLKSTVNVSEFTFHRQKQWD 256
.** :: . *:: *.:: . *:

Supplementary Figure S3. HvGDSL1 protein model. A, B, Protein model of HvGDSL1 (UniProt ID F2DIQ7) predicted by Alphafold (**A**), and predicted by Phyre2 (**B**) based on the crystal structure of *S.cerevisiae* Isoamyl acetate-hydrolizing esterase (c3milA, homology to HvGDSL1: 100% confidence, 90% coverage); Model dimensions (Å): X:72.301 Y:48.693 Z:46.598 **C,** alignment between HvGDSL1 (Query sequence) and c3milA (Template sequence) obtained from Phyre2 showing the known (based on crystal structure) and predicted (based on sequence homology) secondary structure (blue arrow: β-strand, green ribbons, α-helices) of the query and template protein sequence. Orange and red indicate respectively deletion and insertion relative to template. Conserved amino acid are shaded in grey. In the Template Known Secondary Structure letters indicate S = bend, T = hydrogen bonded turn, G = 3-turn helix, B = residue in isolated β-bridge. **D**, Phylogenetic analysis of homologues of HvGDSL1 across representative land plant species. Evolutionary history was inferred by using the Maximum Likelihood method and JTT matrix-based model (Jones et al. 1992). The tree with the highest log likelihood (-7090.03) is shown. Initial tree(s) for the heuristic search were obtained automatically by applying Neighbor-Join and BioNJ algorithms to a matrix of pairwise distances estimated using the JTT model, and then selecting the topology with superior log likelihood value. The tree is drawn to scale, with branch lengths measured in the number of substitutions per site. This analysis involved 24 amino acid sequences and 376 positions. Evolutionary analyses were conducted in MEGA X (Kumar et al. 2018). **E**, Clustal omega alignment of the protein presented in the phylogenetic tree to show conservation of the last 10 amino acids in the C-terminal. Proteins not shown in the alignment do not have this domain. Asterisks indicate a fully conserved residue, colon indicates strongly similar properties, and a period indicates weakly similar properties. The full list and protein identities presented in Supplementary Table S4.

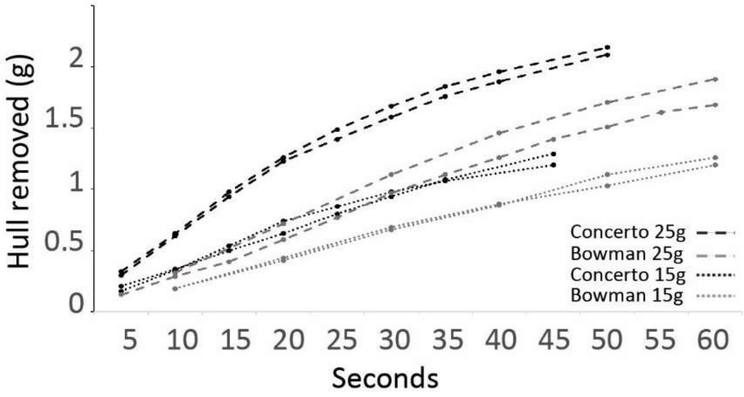

Supplementary Figure S4. Debranner assay optimisation trials. Total weight (g) of husk detached from Bowman (grey) and Concerto (black) grain after different periods (5 to 60 seconds) in the debranner. Quantity of grain tested was 15g (dotted lines) and 25g (dashed lines).

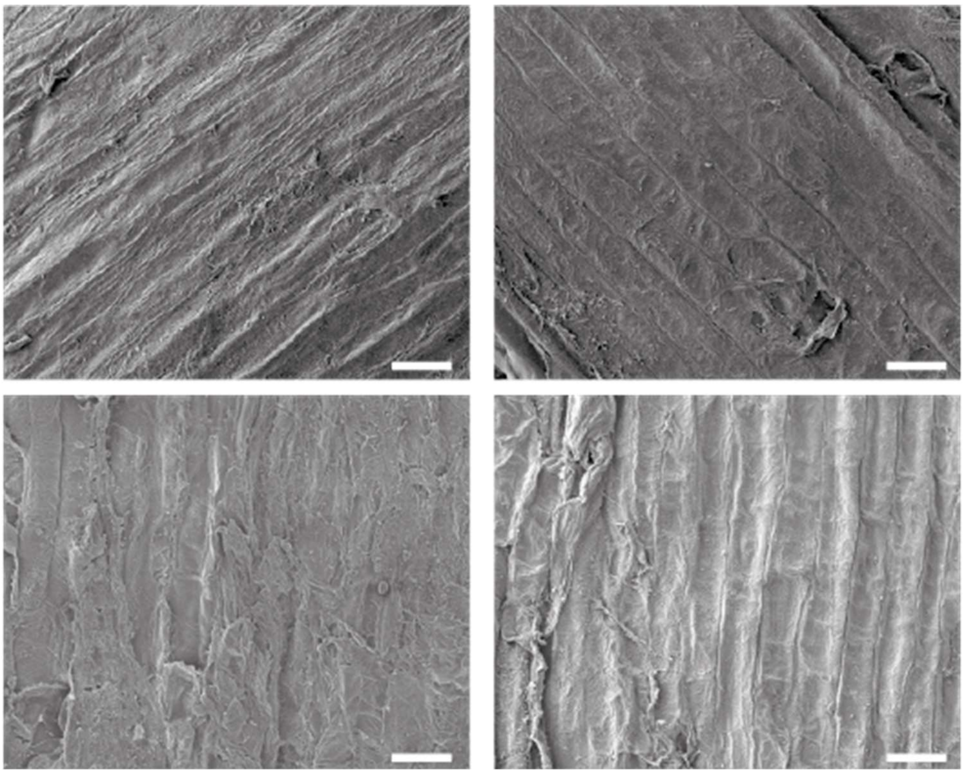

Supplementary Figure S5. Pericarp damage in Bowman caryopses. Scanning electron micrographs of the caryopsis surface following hull removal at 11 days post-anthesis. Images show torn pericarp with reticulated surfaces of underlying layer. Four panels from four independent biological replicates of Bowman grain, all taken at 300 X magnification. Scale bars = 50 µm.
